# Primary Leiomyosarcoma of Gallbladder: A Rare Diagnosis

**DOI:** 10.1155/2012/287012

**Published:** 2012-08-01

**Authors:** Ajay Savlania, Arunanshu Behera, Kim Vaiphei, Harjeet Singh, R. K. Dhiman, Ajay Duseja, Y. K. Chawla

**Affiliations:** ^1^Department of General Surgery, Post Graduate Institute of Medical Education and Research, Chandigarh 160012, India; ^2^Department of Histopathology, Post Graduate Institute of Medical Education and Research, Chandigarh 160012, India; ^3^Department of Hepatology, Post Graduate Institute of Medical Education and Research, Chandigarh 160012, India

## Abstract

Leiomyosarcoma of the gallbladder is a rare entity, constituting about 1.4 per 1000 gallbladder malignancies. Literature review shows female preponderance in sixth decade of life, due to unknown reasons. We report one such rare case of a 50-year-old female admitted with pain in right upper abdomen. On examination, mass was felt in right hypochondrium. The ultrasound abdomen showed mass with loss of interface with liver and cholelithiasis. CECT abdomen showed polypoidal gallbladder malignancy with ill-defined interface with liver. She was operated upon with diagnosis of carcinoma gallbladder; extended cholecystectomy was done. Histopathological examination revealed spindle-cell proliferation and possibility of malignant tumor of mesenchymal origin was kept. This was later confirmed on immunohistochemistry.

## 1. Introduction

Primary leiomyosarcoma of the gallbladder is rarely encountered tumor out of adenocarcinoma and other sarcomatous tumors of gallbladder. These cases constitute about 1.4 per 1000 gallbladder malignancies [1]. Newmark et al. reported the first case in 1879 [2]. Second case reported by Landsteinner in 1904 [3]. In most reported cases, patient's clinical diagnosis was either carcinoma of gallbladder or cholecystitis with cholelithiasis. Preoperative radiological examination may detect the presence of significant lesion but accurate diagnosis is established after histological and immunohistochemical evaluation. Our search through PubMed revealed 20 reported cases of leiomyosarcoma. Therefore, this case is the 21st in this series.

## 2. Case Report

**Figure 1:**
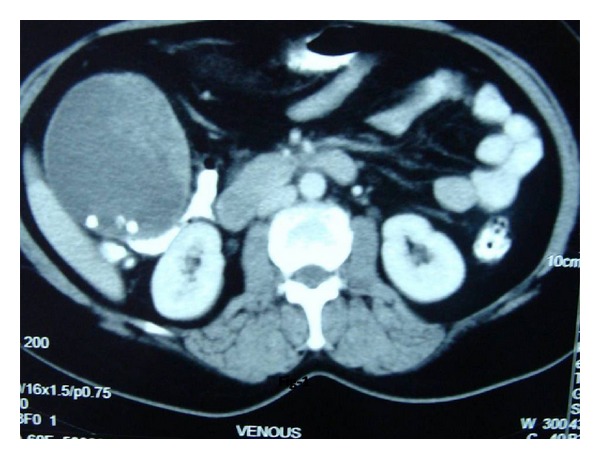
Biphasic CT abdomen showing gallbladder grossly distended: soft tissue mass in gallbladder with multiple intraluminal projections and features suggestive of cholelithasis with polypoidal gallbladder malignancy.

**Figure 2:**
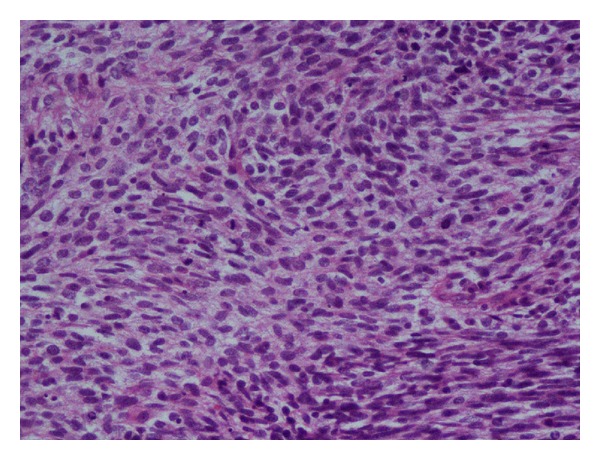
Medium power photomicrograph of the tumor showing moderately pleomorphic round to oval spindle-shaped nuclei with pale cytoplasm and hyperchromatic nuclei. Few scattered bizarre looking nuclei and many atypical mitosis are also noted (H & E, ×250).

**Figure 3:**
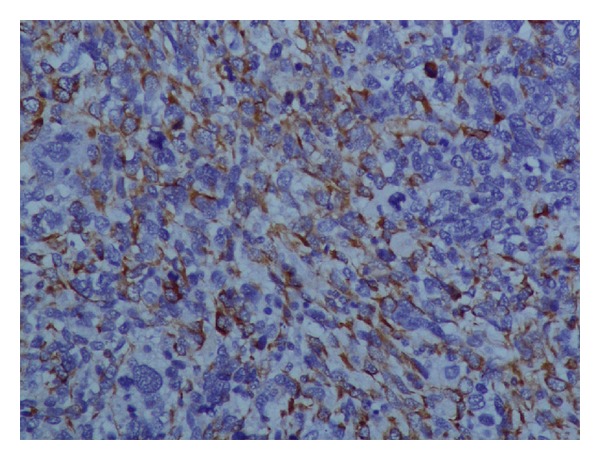
Photomicrograph of immunostained section using smooth muscle actin showing cytoplasmic positivity in almost all the tumor cells (PAP, ×250).

The index patient was a 50-year-old female who presented with history of vague right upper abdominal pain and was found to have a firm palpable mass in right hypochondrium. With a provisional diagnosis of gallbladder cancer, she was subjected to routine investigations including radiological investigations. She had normal haemogram and liver function tests. Ultrasound of the abdomen revealed 9 × 8 cm mass in gallbladder fossa with interface with liver not delineated and cholelithiasis. Biphasic CT abdomen showed gallbladder grossly distended: soft tissue mass in gallbladder with multiple large intraluminal projections and features suggestive of cholelithasis with polypoidal gallbladder malignancy (Figure 1). A provisional diagnosis of carcinoma gallbladder was made. She was subjected to extended cholecystectomy where gallbladder, along with 4b, 5 segments of the liver were resected, and lymph node clearance was done. Intraoperative findings showed patient did not have ascites, liver metastasis, peritoneal, or omental deposits. Porta was free and there was no colonic or duodenal infiltration and no enlarged pericholedochal, paraaortic, or aortocaval lymph nodes. Grossly gallbladder was found to be distended measuring 10 × 6.5 × 4 cm and the lumen was filled with a fleshy friable mass measuring 5 × 4 × 3 cm arising from fundus and body of gallbladder (on cut-section of gallbladder). The tumor was seen extending through the wall of the gallbladder involving the serosa. Rest of the gallbladder mucosa was congested and remaining portions of the body and neck region were thickened. Liver tissue attached measured 6 × 3 × 4 cm. Tiny cystic and pericholedochal lymph nodes were also sampled. Microscopy of the tumor showed spindle to oval to round cells arranged in interlacing fascicles with ulceration of the gallbladder mucosa. Tumor was seen infiltrating transmurally. Tumor cells are moderately pleomorphic with hyperchromasia, occasional bizarre nuclei, multinucleated giant cells, and numerous atypical mitosis (Figure 2). Serosal, cystic duct and liver resection limits were free of tumor. Sections from liver showed focal fatty changes but no tumor deposits. Lymph nodes showed reactive lymphoid hyperplasia. A panel of immunohistochemical markers performed showed strong positivity for antismooth muscle actin (SMA, monoclonal, Dako, Denmark, dilution 1 : 200) (Figure 3), favouring smooth muscle differentiation, negative for pan cytokeratin. Stains performed for CD-117, CD-34, vimentin, and S-100 were noncontributory.

## 3. Discussion

Leiomyosarcoma is a malignant mesenchymal tumor composed of cells showing smooth muscle differentiation. This tumor usually occurs in middle-aged or older individuals. It constitutes significant percentages of retroperitoneal, vascular, extremity, and uterine sarcomas. However, their existence as a mesenchymal neoplasm of gallbladder is still a rare entity except for isolated case reports. Most of the reported cases were females in their sixth decade of life who had vague clinical presentation suggestive of cholecystitis [2, 4]. In the few reports of the radiologic appearances, it has been described as a dilated gallbladder with irregularly thickened wall and polypoid protruding mass into the lumen [2]. The polypoid appearance was present in our case but polypoid appearance of the tumor makes wider differential diagnoses including adenomas or adenocarcinomas and other neoplastic polyps such as cholesterol polyps, inflammatory polyps, and adenomyomatous hyperplasia [5]. Based on ultrasound appearance, the only significant discriminating feature between benign and malignant polypoid lesion is the size with a cut-off figure of 10 mm. False positive and negative results were referred to in the literature with preoperative interpretation as cholecystitis [6]. In the study of Fotiadis et al. [7], the tumor was incidental, missed on ultrasound while the true diagnosis was only made on histological examination. Immunohistochemistry was the most helpful modality to establish the diagnosis. Carefully selected immunohistochemical markers were our strategy in reaching diagnosis within a list of the common mesenchymal tumors that tend to occur in this location. The differential diagnosis of this case essentially includes any spindle-cell tumor of the gallbladder most importantly leiomyosarcoma, fibrosarcoma, undifferentiated carcinoma with spindle cell morphology, carcinosarcoma, and the recently described stromal tumor of the gallbladder with a phenotype similar to that of gastrointestinal stromal tumors (GIST). The undifferentiated carcinoma (spindle-cell or pseudosarcomatous type) as was described by Guo et al. [8] shows positivity for EMA, Keratins, and CEA. Gastrointestinal stromal tumor was presumed to arise from the interstitial cells of Cajal [9]. As its gastrointestinal counterpart, it shows positivity for CD117 (c-kit) and CD34. Our case was negative for both markers. Radical cholecystectomy seems to be the best approach; our patient also underwent radical cholecystectomy. However, many authors recommended palliative surgical bypass due to its poor prognosis, with liver involvement in almost 75% of cases and a five-year survival rate of less than 5%. Adjuvant chemotherapy (doxorubicin, mitomycin C) has prolonged the survival rate in some cases [4, 10, 11]. Patient was in followup for more than one year and patient did not receive any adjuvant therapy.
